# Methylation entropy landscape of Chinese long‐lived individuals reveals lower epigenetic noise related to human healthy aging

**DOI:** 10.1111/acel.14163

**Published:** 2024-04-02

**Authors:** Hao‐Tian Wang, Fu‐Hui Xiao, Zong‐Liang Gao, Li‐Yun Guo, Li‐Qin Yang, Gong‐Hua Li, Qing‐Peng Kong

**Affiliations:** ^1^ Key Laboratory of Genetic Evolution & Animal Models (Chinese Academy of Sciences), Key Laboratory of Healthy Aging Research of Yunnan Province, Kunming Key Laboratory of Healthy Aging Study KIZ/CUHK Joint Laboratory of Bioresources and Molecular Research in Common Diseases, Kunming Institute of Zoology, Chinese Academy of Sciences Kunming China; ^2^ CAS Center for Excellence in Animal Evolution and Genetics Chinese Academy of Sciences Kunming China

**Keywords:** aging, DNA methylation, entropy, epigenetic noise, longevity, long‐lived individuals

## Abstract

The transition from ordered to noisy is a significant epigenetic signature of aging and age‐related disease. As a paradigm of healthy human aging and longevity, long‐lived individuals (LLI, >90 years old) may possess characteristic strategies in coping with the disordered epigenetic regulation. In this study, we constructed high‐resolution blood epigenetic noise landscapes for this cohort by a methylation entropy (ME) method using whole genome bisulfite sequencing (WGBS). Although a universal increase in global ME occurred with chronological age in general control samples, this trend was suppressed in LLIs. Importantly, we identified 38,923 genomic regions with LLI‐specific lower ME (LLI‐specific lower entropy regions, for short, LLI‐specific LERs). These regions were overrepresented in promoters, which likely function in transcriptional noise suppression. Genes associated with LLI‐specific LERs have a considerable impact on SNP‐based heritability of some aging‐related disorders (e.g., asthma and stroke). Furthermore, neutrophil was identified as the primary cell type sustaining LLI‐specific LERs. Our results highlight the stability of epigenetic order in promoters of genes involved with aging and age‐related disorders within LLI epigenomes. This unique epigenetic feature reveals a previously unknown role of epigenetic order maintenance in specific genomic regions of LLIs, which helps open a new avenue on the epigenetic regulation mechanism in human healthy aging and longevity.

## Graphical abstract

Wang et al. constructed high‐resolution methylation entropy (ME) maps in long‐lived individuals (LLIs) using WGBS data. It provides the evidence supporting that a large number of genomic regions, significantly enriched in promoters, displaying significantly lower ME in LLIs (LLI‐specific LERs) than normal aging people. Further analysis revealed a close relationship between these lower entropy regions and age‐related disorders and longevity.

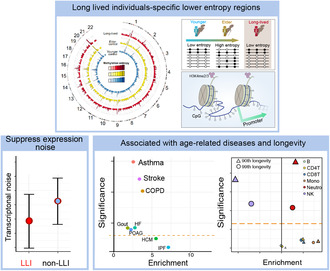

**Abbreviations**
AD: Alzheimer's disease
COPD: chronic obstructive pulmonary disease
CV: coefficient of variation
DNAm: DNA methylation
LER: lower entropy region
LLI: long‐lived individuals
MAV: mean adjusted variability
ME: methylation entropy
MeDIP‐seq: methyl‐DNA immunoprecipitation sequencing
PBMC: peripheral blood mononuclear cell
PD: Parkinson's disease
QE: quantization‐based entropy
S‐LDSC: stratified linkage disequilibrium score regression
WGBS: whole genome bisulfite sequencing

## 1 INTRODUCTION

The aging process is accompanied by the transformation of individual biological systems at various levels (e.g., gene transcription systems) from a well‐organized to noisy state, which may be correlated with dysregulated biological function (Bahar et al., 2006; Cai et al., 2022; Enge et al., 2017; Martinez‐Jimenez et al., 2017). Indeed, a growing body of evidence indicates that disordered gene transcription systems play important roles in driving age‐related diseases, for example, Alzheimer's (AD) (Levy et al., 2020), Parkinson's (PD) (Mar et al., 2011; Schlachetzki et al., 2018), vascular (Zhang et al., 2020), and chronic lung diseases (Angelidis et al., 2019), highlighting the biological significance of transcriptional regulatory system coordination and order in healthy human aging. However, whether a disordered biological system can be modulated during human aging remains largely unknown. As a paradigm of healthy human aging and longevity, long‐lived individuals (LLIs, e.g., centenarians) show the ability to delay serious age‐related diseases (e.g., cardiovascular disease, AD, and cancer) (Evert et al., 2003; Ismail et al., 2016; Newman & Murabito, 2013). Therefore, studies on the LLIs could help decipher the profiles of biological system harmony in successful human aging.

Epigenetic modifications, especially DNA methylation (DNAm), play crucial roles in regulating gene transcription and function in multiple biological processes. The aging process induces significant changes in blood cells at the individual cell type level and in the compositions of cellular compositions (Luo et al., 2022; Mogilenko et al., 2022), and these alterations may manifest in DNAm profiles (Houseman et al., 2015) and contribute to an increased susceptibility to certain chronic diseases, such as heart failure (Ulrich et al., 2024) and asthma (Hoang et al., 2020). Increased stochastically disordered methylation (viz., DNAm noise) across the genome may be caused by epigenetic drift during aging (Fraga et al., 2005; Martin, 2005; West et al., 2013), and the increase in DNAm noise is also associated with progression of age‐related diseases (Landau et al., 2014; Pujadas & Feinberg, 2012; Xie et al., 2011). Shannon entropy, a widely used measure of the degree of uncertainty (Shannon, 1948; Sherwin et al., 2017), has been adopted to characterize age‐related increases in epigenetic noise (Hannum et al., 2013; Sziráki et al., 2018; Wang, Tsui, et al., 2017). Integration of Shannon entropy and one‐dimensional (1‐D) DNAm pattern information obtained from CpG sites in a single consecutive read (viz., DNA sequence) can provide systematic insight into DNAm noise (viz., methylation entropy; ME) (Xie et al., 2011). However, studies on the epigenome of centenarians by whole‐genome bisulfite sequencing (WGBS) (Heyn et al., 2012; Xiao et al., 2023) or methyl‐DNA immunoprecipitation sequencing (MeDIP‐Seq) (Xiao et al., 2015) have mainly focused on average methylation level changes in CpG sites across the genome. Little is known regarding genome‐wide DNAm order in centenarians, which may provide new insights into the epigenetic regulatory system for healthy aging and longevity in humans.

In the current study, we generated the DNAm entropy landscapes of 79 LLIs, 20 elder, and 34 younger control samples. The epigenetic noise landscape for each participant was constructed using WGBS coupled with ME method, and we observed that the LLIs displayed an overall lower‐than‐expected ME level compared to the elder controls. Furthermore, we identified 38,923 genomic regions with LLI‐specific lower ME (termed as LLI‐specific LERs), which were significantly enriched in promoters. Following further analysis, LLI‐specific LERs were found to potentially suppress the transcriptional noise, and genes harboring these regions were preferentially linked to age‐related disorders, suggesting potential functions in the achievement of successful human aging. Taken together, our findings indicate that LLIs possess distinct advantages in balancing epigenetic noise during aging. This study provides a new perspective on the biological characteristics of epigenetic noise during healthy human aging.

## 2 RESULTS

### 2.1 The increase in global methylation noise with age was suppressed in LLIs

**FIGURE 1:**
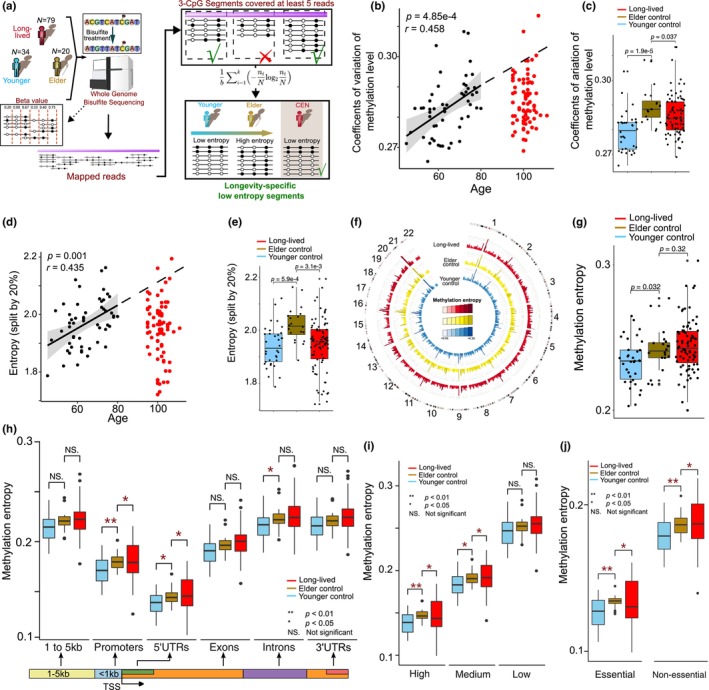
LLIs has lower‐than‐expected methylation entropy in promoters. (a) Schematic diagram of study procedures and DMEAS pipeline to calculate methylation entropy (ME). (b) The methylation coefficients of variation (CVs) among all samples. (c) The methylation CVs in different groups. (d) The methylation quantization‐based entropy (QE) among all samples, while the methylation levels were binned in five categories (i.e., 0%–20%, 20%–40%, 40%–60%, 60%–80%, and 80%–100%). (e) The five catagories' QE in different groups. (f) Landscape of ME in three age groups (i.e., LLI, elder controls, and younger controls) with ME binned in five Mbs. (g) Boxplot showing the global ME of three groups. (h) ME difference in genic regions between three age groups. (i) ME variation in promoters with different CpG_O/E_ among three age groups. (j) ME difference in essential/nonessential gene promoters in three age groups. *p*‐Values of mean tests were determined by two‐sided Kolmogorov–Smirnov test.

We obtained WGBS data (with an average coverage of ~45.2× per sample) of peripheral blood samples, including 133 female individuals (Table 1), including 79 LLIs (>90 years old, 101.0 ± 3.5 years old), 20 elder (>70 years old, 74.4 ± 2.5 years old), and 34 younger controls (45–70 years old, 58.6 ± 5.7 years old), from Hainan Province, China (all the control samples are the spouses of LLIs' children; regarded as natural controls due to their similar living environment to the LLIs) (Figure 1a; Table 1). The methylation level of each detected CpG site was represented as a beta‐value, which ranged from 0 to 1, and then corrected for batch effect. We observed a significant increase in both the genome‐wide methylation coefficient of variation (CV) and Martın‐Plastino‐Rosso (MPR) complexity with age in the control samples. However, LLIs exhibited a significantly lower methylation CV and MPR complexity than the elder controls (Figure 1b,c; Figure S1A,B). The global epigenetic noise of each sample was then assessed using another method based on quantization‐based entropy (QE). Briefly, we divided the CpG sites into five categories by equal‐width binning based on their methylation values and calculated Shannon entropy based on the numbers of CpG sites in each category. In control samples, the QE increased significantly with age, however the QE is lower in LLIs compared to elder controls (Figure 1d,e). The patterns remained stable when the number of categories increased to 10 (Figure S1C,D). Collectively, these results suggest that overall epigenetic noise increases with aging, but this trend is slowed down in LLIs.

**TABLE 1:** Overall population attributes of the cohort.

	LLI	Elder controls (EC)	Younger controls (YC)
Sample size	79	20	34
Age (years)	101.0 ± 3.5	74.4 ± 2.5	58.6 ± 5.7
Biochemistry measurements			
TC (μmol/L)	4.93 ± 1.09	6.04 ± 1.25	5.60 ± 1.67
TG (μmol/L)	1.30 ± 0.54	1.95 ± 1.46	1.72 ± 1.15
HDL (μmol/L)	1.57 ± 0.53	1.39 ± 0.33	1.72 ± 0.24
LDL (μmol/L)	2.69 ± 0.91	3.93 ± 0.87	3.02 ± 1.54
Laboratory blood composition			
WBC (×10^9^/L)	5.78 ± 1.73	7.29 ± 1.31	6.23 ± 1.68
Lymph %	36.49 ± 10.45	36.16 ± 6.69	39.68 ± 10.09
Mid %	7.22 ± 3.94	6.46 ± 2.21	6.42 ± 3.89
Gran %	54.14 ± 10.55	54.74 ± 7.70	53.90 ± 9.89

*Note*: Data are mean ± SD.

Abbreviations: Gran, granulocytes; HDL, high‐density lipoprotein cholesterol; LDL, low‐density lipoprotein cholesterol; Lymph, lymphocyte; Mid, intermediate cell; TC, total cholesterol; TG, total triglyceride; WBC, whole blood cell.

### 2.2 LLIs maintained relative lower ME in promoters

To assess epigenetic noise, we employed ME, an efficient epigenetic computational tool known for dissecting the methylation patterns of contiguous CpG sites (Xie et al., 2011). The genome was divided into small segments containing three consecutive CpG sites during the analyses. After quality control and correction for batch effects (see Material and Methods), 6,057,544 segments with a coverage of ≥5 reads were obtained for ME analyses (Figure 1a). The mean ME in elder controls was significantly higher than younger controls (Figure 1f,g). However, the ME of LLIs was not further significantly increased compared to that of elder controls (who were roughly 26 years younger than LLIs) (Figure 1g), echoing the aforementioned observation of decelerated epigenetic noise increase in the LLIs.

We then assessed the ME status of distinct genic regions as DNA methylation may vary among genome locations. We found that elder controls had significantly higher ME than younger controls in promoters, 5'UTRs, and introns (Figure 1h). However, only in promoters LLIs had significantly lower ME than elder controls (Figure 1h). We also profiled ME status in intergenic genomic elements, including four types of transposable elements (SINE, LINE, LTR, and DNA repeat), enhancer, and insulator. No significant ME differences were observed among three groups in these elements (Figure S3). In addition, many studies have shown that DNA methylation varies in promoters with varied CpG content, which biases regulatory activity (Nguyen et al., 2016; Saxonov et al., 2006). Therefore, we investigated the relationship between ME and promoter CpG content. We classified promoters into three categories according to their observed/expected CpG content ratios (CpG_O/E_) and observed that promoter ME is positively associated with CpG_O/E_ ratios (Figure 1i). Only in high CpG_O/E_ promoters did LLIs sustain a lower ME status than the elder, although ME in such promoters is significantly higher in the elder compared to younger controls (Figure 1i). As increased epigenetic noise may impair the gene expression concordance and generally considered to be deleterious (Pujadas & Feinberg, 2012; Wang & Zhang, 2011), we proposed that the ME should decrease as gene importance increases (Figure S4A). We obtained a list of 1842 essential genes (see Section 5.5) and found that elder controls had higher ME in promoters of both essential and nonessential genes than younger controls. Interestingly, although LLIs had higher ME in nonessential gene promoters than the elder controls, they sustained significantly lower ME in essential gene promoters than the elder controls (Figure 1j). For example, the essential gene DNA Polymerase Delta 2 (*POLD2*), which participates in high fidelity genome replication and DNA repair (Fuchs et al., 2021), showed decreased ME in LLIs than elder controls (Figure S4B).

### 2.3 LLIs possessed significant numbers of genomic regions with decreased ME

**FIGURE 2:**
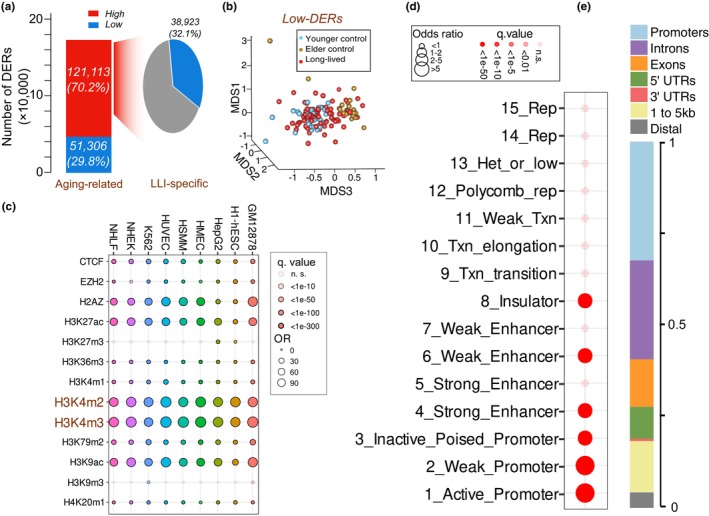
Analysis of differential entropy in CpG segments among the LLIs, elderly, and younger controls. (a) Distributions of aging‐related DERs and LLI‐specific LERs. (b) First three coordinates of the multidimensional scaling (MDS) representation of the LLIs and control samples based on ME levels of LLI‐specific LERs. (c) Enrichment results of LLI‐specific LERs over histone modification peaks of nine cell lines. Circle darker shades indicate more significant *p* values, and sizes are reflective of odds ratio (OR). (d) Enrichment results of LLI‐specific LERs over GM12878 ChromHMM annotations. Circle darker shades indicate more significant *p* values, and sizes are reflective of odds ratio (OR). (e) Distribution of LLI‐specific LERs annotated in genic regions.

To decipher LLI‐specific ME characteristics across the epigenome, we analyzed the differences in ME in all 6,057,544 segments among the three groups. First, we deconvoluted main blood cell type compositions from the methylomes by robust partial correlation method (Teschendorff et al., 2017), and used the estimated cell proportions to adjust the effect of cell heterogeneity. Using a threshold of FDR <0.05, we identified 172,419 genomic segments with different entropy (hereafter defined as differential entropy regions, DERs) between the elder and younger controls (considered as the aging‐related DERs). Results showed that the vast majority (121,113; 70.2%) of aging‐related DERs displayed higher ME (high‐DERs) in elder controls compared to younger controls, in accordance with the genome‐wide increase in entropy pattern during aging. Given that multiple studies have shown that LLIs typically maintain a younger epigenetic status (e.g., younger epigenetic age) (Komaki et al., 2023; Xiao et al., 2023), we then asked whether some of these aging‐related high‐DERs were maintained at a younger epigenetic state in LLIs, that is, some aging‐related DERs had lower‐than‐expected ME in LLIs. Indeed, of the 121,113 aging‐related high‐DERs, we identified 38,923 (32.1%) exhibited opposite ME directions to the aging‐related changes in LLIs (i.e., lower ME in LLIs compared with elder control samples) (Figure 2a; Table S2). Thus, these 38,923 regions could be characterized as LLI‐specific lower entropy regions (LLI‐specific LERs). By visualizing the inter‐sample pairwise distance based on LLI‐specific LERs using multidimensional scaling (MDS) analysis, we noticed that the LLI samples were closer to younger controls and further away from elder controls (Figure 2b; Figure S5).

We then looked into the biological implication of the LLI‐specific LERs, first characterizing their chromatin state using histone modification markers. We collected the information of chromatin states annotated by ChIP‐Seq data for 12 histones and one canonical chromosome protein CTCF, in nine cell lines from different tissues, including blood, skin, liver, and embryonic stem cells. We observed that the LLI‐specific LERs were greatly overrepresented in activated promoter markers (viz., H3K4me2 and H3K4me3) (Bernstein et al., 2005; Santos‐Rosa et al., 2002) (Figure 2c). Likewise, we noticed that LLI‐specific LERs displayed a substantial enrichment in regions with promoter activity, according to the predicted annotation by the ChromHMM standard 15 chromatin states in B‐lymphoblastoid cells (GM12878) (Figure 2d). When we annotated the LLI‐specific LERs to genic regions, we found that 32.4% of them were located in promoters (Figure 2e), which was significantly higher than the expected ratio of 6.62% (estimated by all 6,057,544 segments, OR = 6.78, Fisher's exact test *p* < 2.20 × 10^−16^). This result differs from the observation based on the traditional methods by analyzing methylation levels, with the predominant signal being LLI‐specific hypermethylated CpGs overrepresented in heterochromatin region (Figure S2) (Xiao et al., 2023).

**FIGURE 3:**
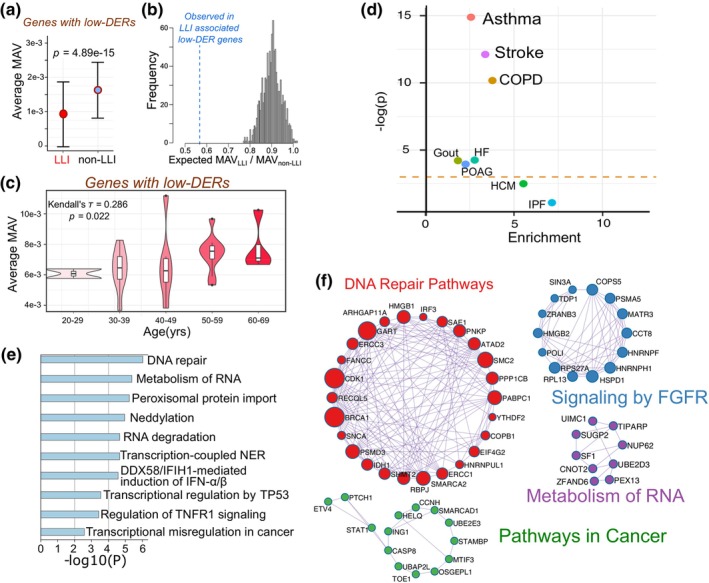
The potential biological function of LLI‐specific LERs. (a) Mean‐adjusted expression variances of genes whose promoters containing LLI‐specific LERs in LLIs and controls. *p*‐Values were calculated by two‐sided Wilcoxon's rank sum test. (b) Frequency distributions of MAV_LLI_/MAV_non‐LLI_ ratio from 1000 bootstrap sets of random selected genes. (c) Mean‐adjusted expression variances of genes whose promoters containing LLI‐specific LERs differs in different age groups. (d) Results from stratified LD score regression (S‐LDSC) applied to genes whose promoters containing LLI‐specific LERs to partition aging‐related traits heritability within genome. Dashed horizontal line represents the *p*‐value threshold of 0.05. (e) Top 10 enriched gene sets of genes whose promoters containing LLI‐specific LERs. (f) Protein–protein interaction (PPI) networks constructed based on genes whose promoters containing LLI‐specific LERs. Node size represents the degree centrality of each gene.

The significant enrichment pattern in promoters hint us to investigate whether such LLI‐specific LERs influence gene expression. Given previous studies indicating a direction relationship between methylation entropy and gene expression variability (i.e., transcriptional noise) (Fang et al., 2023), we analyzed single cell RNA‐Seq data to see if LLI‐specific LERs located in promoters contribute to the reduction of cell‐to‐cell expression variability in genes. We collected single cell transcriptome data of peripheral blood mononuclear cell (PBMC) from non‐LLIs with five different age groups (Komaki et al., 2023) and a cohort containing seven LLIs and five controls (Bernstein et al., 2005). As expected, genes with LLI‐specific LERs in their promoters had significantly lower mean adjusted variability (MAV) in LLIs (MAV_LLI_) as compared to non‐LLI controls (MAV_non‐LLI_) (Figure 3a). The mean MAV_LLI_/MAV_non‐LLI_ ratio of the genes with LLI‐specific LERs in promoters was significantly lower than that of genes lacking such DERs (*p* < 0.001, Figure 3b). Yet, MAV of these genes increased with age in non‐LLI people (Figure 3c), which is consistent with the fact that ME in LLI‐specific LERs is positively correlated with non‐LLIs' age.

### 2.4 LLI‐specific LERs exhibited high potential in healthy aging

To further investigate the potential role of LLI‐specific entropy decrease, we then evaluated the contribution of genes with LLI‐specific LERs in promoters to the SNP‐based heritability of aging phenotypes. We adopted the stratified linkage disequilibrium score regression (S‐LDSC) tool to partition heritability of eight aging‐related phenotypes (including heart failure, idiopathic pulmonary fibrosis, and stroke) into gene sets whose promoters containing LLI‐specific LERs using GWAS summary statistics (Zhou et al., 2022). In our analysis, asthma heritability was the most significantly enriched in such genes, followed by stroke and chronic obstructive pulmonary disease (COPD) (Figure 3d). Our findings indicated a considerable association between LLI‐specific LERs and aging‐related disorders.

We next performed functional enrichment analysis to explore the potential biological functional roles/pathways regulated by LLI‐specific LERs. Results showed that genes with LLI‐specific LERs in promoters were significantly enriched in series of biological processes or pathways such as DNA repair, metabolism of RNA, peroxisomal protein import, and transcriptional regulation by *TP53* (Figure 3e). Protein–protein interaction analysis of these genes showed four networks that were overrepresented in DNA repair pathways, pathways in cancer, signaling by FGFR, and metabolism of RNA (Figure 3f). These results could aid in functional interpretation of the LLI‐specific lower ME.

### 2.5 Numerous LLI‐specific LERs were neutrophil‐specific, exhibiting high potential in healthy aging

**FIGURE 4:**
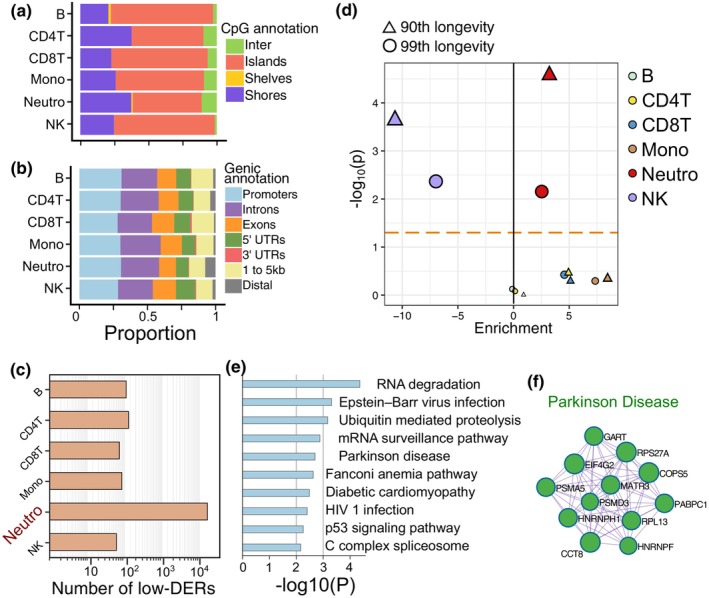
Neutrophil is the primary cell type harboring LLI‐specific LERs. (a) Proportions of genic annotations in each cell type‐specific LLI‐specific LERs. (b) Proportions of CpG island annotations in each cell type‐specific LLI‐specific LERs. (c) Counts of six kinds of cell type‐specific LLI‐specific LERs, the y axis is log‐transformed. (d) Results from stratified LD score regression (S‐LDSC) applied to genes whose promoters containing cell type‐specific LLI‐specific LERs to partition longevity heritability within genome. “99th longevity” and “90th longevity” denotes cases surviving at or beyond the age corresponding to the 99th/90th survival percentile, respectively. The dashed horizontal line represents the *p*‐value threshold of 0.05. (e) Top 10 enriched gene sets of genes whose promoters containing neutrophil‐specific LLI‐specific LERs. (f) Genes whose promoters containing neutrophil‐specific LLI‐specific LERs formed a Parkinson disease‐related PPI network.

To explore the association between the six main blood cell types and the existence of LLI‐specific ME decrease, we identified cell type‐specific LLI‐specific LERs by CellDMC algorithm, which performs interaction analysis between estimated cell proportions and phenotypes (Zheng et al., 2018). A significant decrease in B‐cell and CD4^+^ T‐cell proportion was observed in LLIs (Figure S6A), and 43.1% of (16,762/38,923) the LLI‐specific LERs occurred in specific cell types (Table S3). All six types of cell type‐specific LLI‐specific LERs were overrepresented in CpG islands and promoters (ranges from 28% to 31% Figure 4a,b). We found neutrophils accounting for 97.3% (16,308/16,762) of this cell type‐specific low‐DERs (Figure 4c), although its proportions did not differ significantly between LLIs and elder controls (Figure S6A).

To further investigate the contribution of low ME cell state signatures to the longevity, we utilized the S‐LDSC to partition heritability of longevity to gene sets using GWAS summary statistics (Deelen et al., 2019). Our results showed that longevity heritability was only significantly enriched in genes harboring neutrophil‐specific LLI‐specific LERs in promoters (Figure 4d). We next conducted functional enrichment analysis for the neutrophil‐specific LLI‐specific LERs. Results showed that genes with the low‐DERs in promoters were significantly enriched in pathways like RNA degradation, ubiquitin mediated proteolysis, and diabetic cardiomyopathy, p53 signaling pathway (Figure 4e). Protein–protein interaction analysis of these genes revealed a network that is overrepresented in Parkinson disease (Figure 4f). These results suggest that decreased ME in genomic regions of neutrophil may contribute to the healthy aging of LLIs.

## 3 DISCUSSION

In this study, we observed an overall epigenetic noise increased with aging, a trend that is notably attenuated in LLIs. On a genome‐wide scale, this may be associated with a more concentrated methylation level distribution in LLIs (Figure S7). We next constructed and compared the DNA ME landscapes of LLIs and matched controls with varying ages, aiming to decipher the characteristics of epigenetic noise involved in healthy human aging and longevity. Overall, the general control samples showed an age‐related increase in global epigenetic noise, which was in line with previous studies (Hannum et al., 2013; Sziráki et al., 2018; Wang, Tsui, et al., 2017) and likely indicated the accumulation of genomic damage (Gladyshev, 2016) and epigenetic modification system dysregulation during aging (Xiao et al., 2019). Interestingly, we found that the increase trend of entropy was suppressed, to some extent, in LLI epigenomes, suggesting that they deviated from the aging trend, echoing the observation of younger DNAm states for longevity people in the whole genome DNA methylation profiling studies (Komaki et al., 2023; Xiao et al., 2023).

Importantly, we also found that some specific genomic regions display an increase in ME during aging in non‐LLIs (i.e., elder vs. younger controls) but present a significant decrease ME in LLIs compared to elder controls (i.e., LLI‐specific LERs). These regions were overrepresented in promoters and favored over those with higher CpG densities. The results likely suggest that LLIs prefer to maintain a less noisy epigenetic status in genomic regions with more significant biological roles, as promoter activity is significantly correlated with CpG content (Hartl et al., 2019). Given that the DNA methylation heterogeneity at promoters is largely caused by aging‐related stochastic methylation gain (Senapati et al., 2023), the overrepresentation of LLI‐specific LERs on promoters indicates that the imprecise epigenetic inheritance systems (Ming et al., 2021; Wang et al., 2020) may have undergone some compensation in the long‐lived people. Correspondingly, we also observed that LLIs kept lower transcriptional noise in genes with LLI‐specific LERs, implying that the decreased ME may be the epigenetic factor mediating selective pressure on gene expression noise (Puzović et al., 2023). Among the genes with LLI‐specific LERs, only 22% (47 out of 215) were previously reported as upregulated in LLIs (Table S4) (Karagiannis et al., 2023; Wu et al., 2023), suggesting the association between LLI‐specific LERs and highly expressed genes in LLIs is rather weak. These findings altogether point to the likelihood that LLIs preserve the epigenetic order of certain genomic regions with biological roles particularly in the regulation of gene transcription.

We then explored the cell‐type specific LLI‐specific LERs since ME reduction in different cell types may serve diverse biological functions. Indeed, several GO terms were exclusively enriched in LLI‐specific LERs from particular cell types (Figure S6B), such as mTOR signaling pathway in natural killer (NK) cells and TP53 activity in CD4^+^ T cells. Using deconvolution analysis, we showed that LLI‐specific LERs were primarily associated with neutrophils. This suggests that certain LLI‐specific LERs mainly exists in specific blood cell types, while others are present in a broader range of blood cell types. A growing body of evidence has shown that neutrophil heterogeneity may arise from organismal aging in accompany with significant downregulation of DNA methylation/demethylation machinery (Casanova‐Acebes et al., 2013; Lu et al., 2021). Previous study also showed that such age‐induced neutrophil alterations in the circulation are detrimental for vascular (Adrover et al., 2019; Gullotta et al., 2023), pointing to the potential benefit of the neutrophil low‐DERs in maintaining vascular health. The disparities in the gene functional enrichments between LLI‐specific LERs and neutrophil‐specific LLI‐specific LERs suggested that methylation homeostasis in specific cells may function on specialized pathways. For example, we identified a network of genes associated with Parkinson's disease (PD) from neutrophil‐specific LLI‐specific LERs, whereas this network was not identified from whole blood LLI‐specific LERs. Given that neutrophils contribute to the significantly increased epigenetic age observed in Parkinson's disease (PD) patients (Horvath & Ritz, 2015), it is conceivable that the specific reduction in ME of certain genes in neutrophil‐specific may aid LLIs in evading the onset of PD. Collectively, these results highlight the role of epigenetic order maintenance in neutrophil in healthy human aging and longevity, which may provide a new insight into the age‐related disease avoidance in LLIs.

## 4 CONCLUSION

By deciphering the epigenetic noise landscapes of LLIs and control cohorts, we found that there are a large number of genomic regions displaying lower‐than‐expected methylation entropy in LLIs. Multiple lines of evidence support that such LLI‐specific feature in entropy decrease is unlikely to be random event but tightly associated to the health‐related signaling pathways and the aging‐related traits. Thus, our study points to a hitherto unrecognized role for maintaining epigenetic order of some specific genomic regions in LLIs and most plausibly in human healthy aging. Further functional study on these regions would help open a new avenue on the epigenetic regulation mechanism in human healthy aging and longevity.

### 4.1 Limitation

Notably, this study is limited by the fact that we did not include the epigenome of male participants, who were not available in our cohort. Therefore, it is still open question whether the methylation entropy status we observed in female are also manifested in male, or if these characteristics are sex‐specific. Our current results were primarily derived from high‐throughput sequencing data, and future study incorporating experimental validation may offer some new insights into the mechanism underlying the observed methylation entropy pattern in LLIs.

## 5 MATERIALS AND METHODS

### 5.1 Sampling and sequencing

Peripheral blood samples were obtained from 133 female individuals, including 79 LLIs (>90 years old) and 54 F1 offspring spouses (F1SPs) of LLIs, from Hainan Province, China (Table S1). We selected F1SP as controls to reduce the effects of environments on the analyses as much as possible, as they shared very similar living conditions and even diets with the long‐lived individuals for a long time. This strategy of control selection that has been widely used in previous longevity population studies (Passtoors et al., 2013; Ryu et al., 2021; Xiao et al., 2018, 2022, 2023). These controls were divided into younger (45–70 years old) and elder (>70 years old) groups. We also assayed the proportions of three main white cell types (i. e., lymphocytes, minimum inhibitory dilution cells, and neutrophils) for each sample.

Genomic DNA was extracted by from the blood samples with AxyPrep™ Blood Genomic DNA Maxiprep kits (Axygen Biosciences, USA). The DNA was fragmented by sonication using a Covaris S2 Ultrasonicator system (Covaris Inc., USA). Downstream library preparation was performed using an EZ DNA Methylation‐Gold kit (Zymo Research, USA) according to manufacturer's instructions. The WGBS libraries were sequenced on an Illumina HiSeq 4000 platform (Illumina, USA) in paired‐end mode with 150 bp. All participants provided written informed consent for their participation in this study and all protocols were approved by the Ethics Committee at the Kunming Institute of Zoology, Chinese Academy of Sciences. WGBS data of 111 samples were previous published (Xiao et al., 2023) and collected from Genome Sequence Archive (Wang, Song, et al., 2017) in National Genomics Data Center (CNCB‐NGDC Members and Partners, 2021), China National Center for Bioinformation/Beijing Institute of Genomics, Chinese Academy of Sciences, under accession number HRA000502/HRA003301 at https://bigd.big.ac.cn/gsa‐human. Additionally, WGBS data for 22 samples were newly generated and have been deposited under accession number HRA006665.

### 5.2 WGBS read alignment and CpG methylome profiling

The quality control procedures for sequencing reads were performed using the FastQC v0.11.6 program (http://www.bioinformatics.babraham.ac.uk/projects/fastqc). We retained reads with Q20 > 90% and Q30 > 85% for downstream analysis. We then mapped the reads to the human reference genome NCBI GRCh37 using Bismark v0.17.0 (Krueger & Andrews, 2011) with default parameters. Bowtie2 v2.1.0 (Langmead & Salzberg, 2012) was called by Bismark for read alignment.

The methylation level of each CpG site was represented as follows:

$$\text{Methylation level}=\frac{N_{c}}{N_{c}+N_{T}}$$

where *N*_*C*_ is the number of reads containing methylated cytosine and *N*_T_ is the number of reads containing unmethylated cytosine. Methylation levels were extracted by bismark_methylation_extractor script in Bismark using “‐‐comprehensive ‐‐counts ‐‐bedGraph ‐‐cytosine report.” Among the CpG cytosines, we used a cutoff of at least 10× coverage in over 60% of the 133 samples and a total of 21,550,709 CpG cytosines were used for estimation of coefficients of variation (CVs) and quantization‐based entropy analysis. We corrected the batch effect among methylation levels by the removeBatchEffect function in limma R package (Ritchie et al., 2015). Differentially methylated CpGs (DMCs) were identified by R package methylKit with function calcDiffMeth. The thresholds of DMC were *Q*‐value <0.01 and absolute methylation level difference >0.1.

### 5.3 Calculation of metrics for noise

We introduced several calculation procedures for the metrics used in this study. Here, CV was calculated as follows:

$${\mathrm{CV}}_{i}=\frac{\sigma_{i}}{\mu_{i}}$$

where *CV*_*i*_ is the *CV* of sample *i*; *σ*_*i*_ is the standard deviation of 21,550,709 CpG cytosine methylation values of sample *i*; and *μ*_*i*_ is the mean of the CpG cytosine methylation values of sample *i*. We calculated MPR complexity by R package statcomp v 0.1.0.

Quantization‐based entropy value was calculated as Shannon entropy:

$$\text{Entropy}=\sum_{i=1}^{k}\left(-\frac{n_{i}}{N}\log_{2}\;\frac{n_{i}}{N}\right)$$

where *k* (= 5 or 10 in this study) is the number of equal‐width bins used to discretize methylation values; *N* is 21,550,709; and *n*_*i*_ is the number of CpG cytosines whose methylation levels fall into *i*‐th bin, whose range is ((*i*‐1) × 10%, *i* × 10%). For example, if *k* = 10 and *i* = 5, then *n*_*i*_ is the number of cytosines with methylation values >40% and ≤ 50%; when *i* equals 1, *n*_*i*_ is the number of cytosines with methylation values ≥0% and ≤ 10%.

### 5.4 Methylation entropy (ME) calculation

ME was calculated using DNA methylation entropy analysis software (DMEAS) (He et al., 2013). Referred to published study for methylation heterogeneity estimation tool development (Lee et al., 2023), we selected DNA segments with three consecutive CpGs, which were covered by at least five reads observed in at least half of the three groups (i.e., 40 in long‐lived individuals, 17 in younger controls, and 10 in elder controls). Here, Shannon entropy was calculated as follows:

$$\text{Entropy}=\frac{1}{b}\sum_{i=1}^{k}\left(-\frac{n_{i}}{N}\log_{2}\;\frac{n_{i}}{N}\right)$$

where *b* is the number of consecutive CpG sites in a segment (i.e., *b* = 3) and *k* is the number of observed methylation patterns in this segment (1 ≤ *k* ≤ 8); *N* is the total number of reads covering this segment and *n*_*i*_ is the number of reads matching methylation pattern *i* (1 ≤ *i* ≤ N). The DMEAS input files were the CpG_context files generated by Bismark, which were split by chromosomes using in‐house script. The methylation level of a segment was defined as the percentage of methylated CpG cytosines observed. We also ruled out batch effects by the removeBatchEffect function implemented in limma R package. After preprocessing, there is no significant batch effects between different batches (Figure S1E).

### 5.5 Genome annotation

The histone modification annotations and chromatin states were retrieved from the UCSC genome browser (Ernst et al., 2011; Ram et al., 2011). Histone modifications were annotated using Broad Histone of nine cell types. Chromatin states were from GM12878 ChromHMM segmentation because this cell line was isolated from the peripheral blood of a female as that in our study. Genic regions and distal elements were annotated using the R package annotatr v1.16.0, with the R package TxDb.Hsapiens.UCSC.hg19.knownGene database. Promoter classification based on CpG_O/E_ was performed referring to Zeng & Yi (2010), and promoters were binned into three classes (high, medium, and low) according to 1/3 and 2/3 quantiles of CpG_O/E_. Gene essentiality data were obtained from the OGEE v3 database (Gurumayum et al., 2021), and we retained 1842 genes which were identified as hematopoietic and lymphoid essential genes in at least 10 experiments.

### 5.6 Differential analysis of ME

Six blood cell type compositions (i.e., B cells, CD4+ T cells, CD8+ T cells, monocytes, neutrophil, and natural killer cells) were first estimated from methylome by R package EpiDISH with the robust partial correlation deconvolution method (Teschendorff et al., 2017). Differential methylation entropy regions (DER) between groups were identified using the partial Kendall's correlation analysis with R package ppcor, including the six cell compositions as covariates. DERs were considered significant at false discovery rates (FDR) below 0.01. In total, 38,923 DERs were identified as LLI‐specific lower entropy regions. *CellDMC* function (Zheng et al., 2018) in EpiDISH were used to estimate the specific cell type(s) where the ME changes occur.

### 5.7 Enrichment analysis

ChromHMM and histone enrichment analyses of DERs were performed using hypergeometric distribution to calculate significance with the R package LOLA v1.18.0 (Sheffield & Bock, 2016). Functional enrichment analysis was performed using Metascape (Zhou et al., 2019), where we only considered genes whose promoters contained ≥3 low DERs.

### 5.8 Transcriptional noise analysis based on single‐cell RNA‐Seq data

Two publicly available PBMC scRNA‐Seq datasets were retrieved for transcriptional noise estimation: (1) a study conducted in subjects with different ages (hereafter as “aging study”) (van der Wijst et al., 2018), and (2) a longevity study conducted in seven LLIs and five non‐LLI controls (hereafter as “longevity study”) (Hashimoto et al., 2019). In both datasets, cells (1) with ≥500 expressed genes with non‐zero read counts and (2) with ≤5% mitochondrial proportions were retained. The read counts were log‐normalized using *NormalizeData* function in Seurat v4.1.1. The transcriptional noise was calculated by gene expression mean‐adjusted variability (MAV) referring to a recent methylation entropy study (Fang et al., 2017).

### 5.9 S‐LDSC analysis

LDSC v1.0.1 were used to partitioned heritability analysis of longevity and aging‐related traits (Finucane et al., 2015). Summary statistics data of eight traits from Eastern Asian (EAS) subjects were retrieved from Global Biobank Meta‐analysis Initiative (GBMI, https://www.globalbiobankmeta.org/resources). Longevity meta‐analysis summary statistics for European (EUR) ancestry population were retrieved from GWAS catalog (https://www.ebi.ac.uk/gwas, accession: GCST008598). The transcribed regions of genes containing LLI‐specific LERs were used as the gene sets, and the LD score window was 1cM (default). The 1000 Genomes (1000G) Phase 3 EAS and EUR data files (downloaded from https://zenodo.org/record/7768714) were used for eight EAS traits and longevity separately.

### 5.10 Statistical analysis and data visualization

The statistical tests (e.g., Fisher's exact test, hypergeometric test, and Kolmogorov–Smirnov test) are provided in the figure legends. In each boxplot, the center horizontal line is the median value and outliers are not shown. Three dimensional projections of DERs were generated by top 20,000 variable DERs using multidimensional scaling (MDS). Most panels were drawn by R v4.2.3 and modified later. Circos plots were drawn by Circos v0.69‐8.

### 5.11 Role of the funding source

The funders of the study had no role in study design, data collection, data analysis, data interpretation, or writing of the report.

## AUTHOR CONTRIBUTIONS

QPK and FHX concepted and designed this study. HTW and FHX analyzed the data. HTW, FHX, ZLG, and LQY contributed to the sampling. ZLG, LYG, and HTW did the laboratorial work. GHL assisted with interpretation of results. QPK, HTW, and FHX had access to the data. HTW and FHX had verified the data. HTW, QPK, and FHX wrote the draft. QPK and FHX supervised this study. All authors read and approved the final manuscript.

## 6 FUNDING INFORMATION

This study was supported by National Key R&D Program of China (2023YFC3603400), CAS Project for Young Scientists in Basic Research (YSBR‐076), West Light Foundation (to F.‐H.X.) of the Chinese Academy of Sciences, National Natural Science Foundation of China (82371580, 82071595), Yunnan Fundamental Research Projects (202101AS070058, 202201AS070080, 202301AT070281, 202401AW070011), High‐level Talent Promotion and Training Project of Kunming (Spring City Plan; 2020SCP001), Yunling Scholar of Yunnan Province, Reserve Talent Project of Young and Middle‐aged Academic and Technical Leaders in Yunnan Province (202305AC160029).

## CONFLICT OF INTEREST STATEMENT

All authors declare no competing interests.

## Supporting information


Figures S1–S7

Tables S1–S4

## Data Availability

All raw sequence data in this paper have been deposited in the Genome Sequence Archive in National Genomics Data Center, China National Center for Bioinformation/Beijing Institute of Genomics, Chinese Academy of Sciences, under accession number HRA000502/HRA003301/HRA006655 at https://bigd.big.ac.cn/gsa‐human. In‐house scripts for DER identification and figure generation were deposited in GitHub (https://github.com/Konglab404/ME).
