# Supplementary material for: Methylation entropy landscape of Chinese long‐lived individuals reveals lower epigenetic noise related to human healthy aging
Source: Aging Cell. 2024 Apr 2;23(7):e14163. doi: 10.1111/acel.14163 (PMC11258444; doi:10.1111/acel.14163)
Supplement: Supplementary file 1 — Figures S1–S7 [file ACEL-23-e14163-s002.docx]

**Supplementary Tables**

**Table S1** WGBS sequencing and mapping statistics

**Table S2** LLI-specific lower entropy regions (LERs)

**Table S3** Cell type-specific LLI-specific LERs identified by *CellDMC*

**Table S4** List of genes sustaining LLI-specific LERs.

**Supplementary Figure Legends**


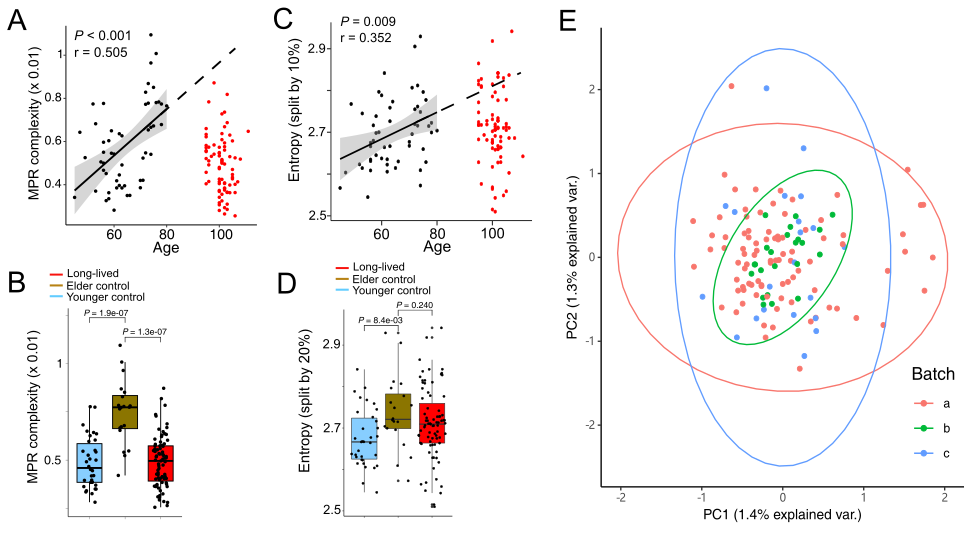


**Figure S1**. **Increased epigenetic noise with aging.** (**A**) The methylation MPR complexity of all samples. (**B**) The methylation MPR complexity in different groups. (**C**) The methylation quantization-based entropy (QE) among all samples, while the methylation levels were binned in 10 categories. (**D**) The 5-catagories QE in different groups. The correlation relationships between epigenetic noise metrics and age were tested by Pearson’s correlation test. The P-values in panels **B** was calculated by two-sided Kolmogorov-Smirnov test. (**C**) PCA plot of ME status in different batches.


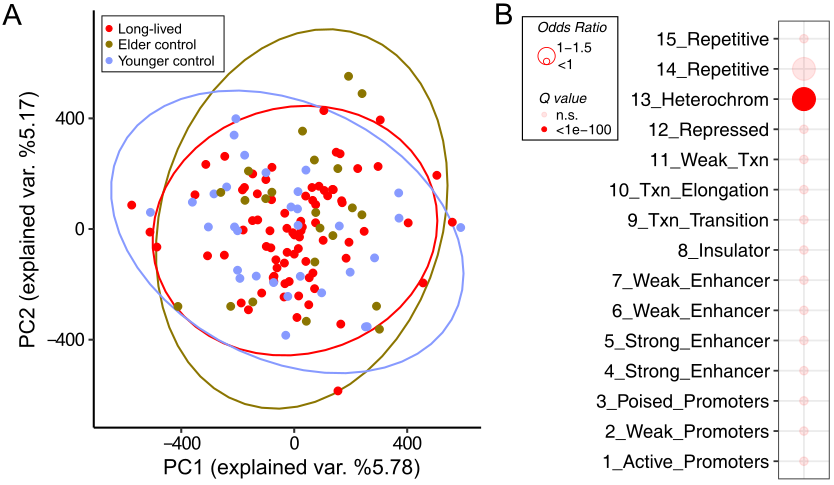


**Figure S2**. **Differential methylated CpG (DMC) analysis.** (**A**) The PCA based on methylation levels of 133 samples. (**B**) Enrichment results of LLI-specific hyper-DMCs over GM12878 ChromHMM annotations.


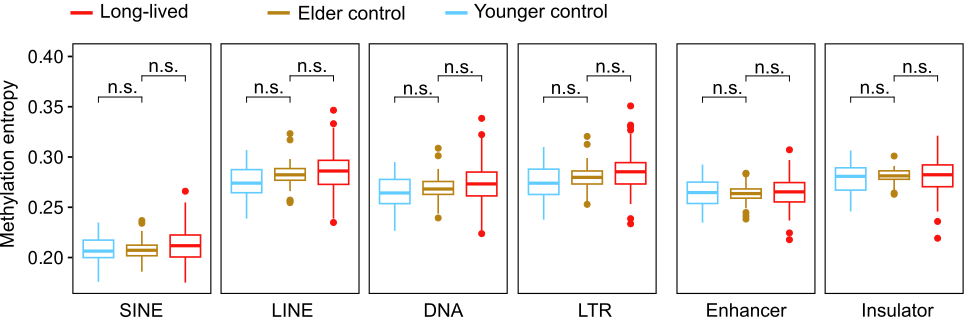


**Figure S3**. **ME distribution in transposable elements, enhancers, and insulators among three age groups.**


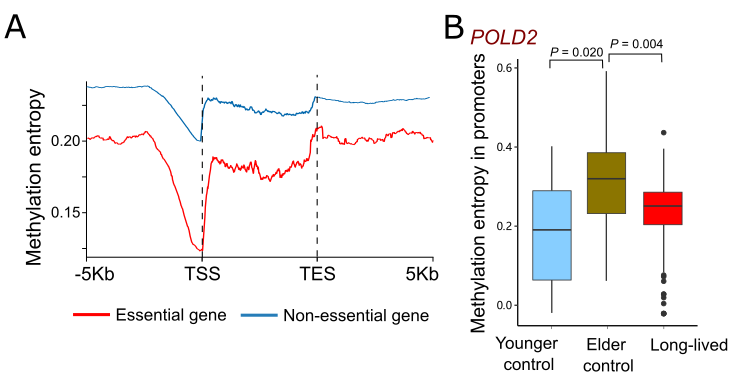


**Figure S4**. **(A)** LLI’s ME distribution in essential/non-essential genes, suggesting the negative correlation between gene importance and ME. (**B**) Promoter ME of *POLD2* among three groups.


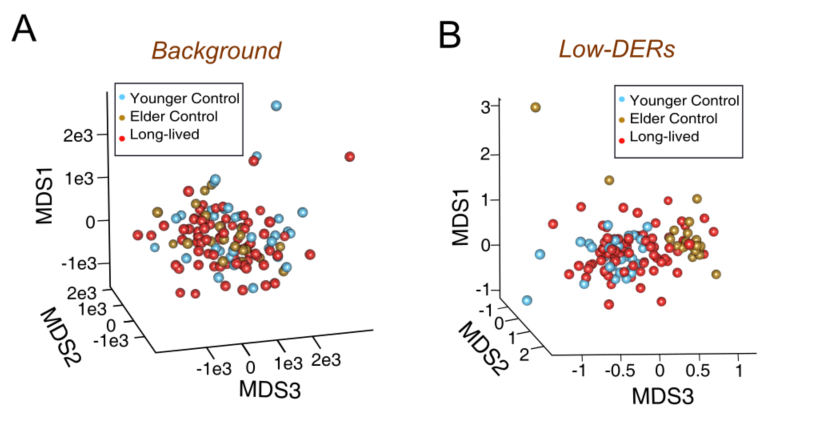


**Figure S5**. **MDS analysis showed that LLI-specific LERs sustained a relative younger epigenetic state.** (**A**) The MDS dimensional reduction results based on all 6,057,544 segments. (**B**) The MDS dimensional reduction results based on top 20,000 LLI-specific LERs with higher ME variances. First three coordinates were shown.


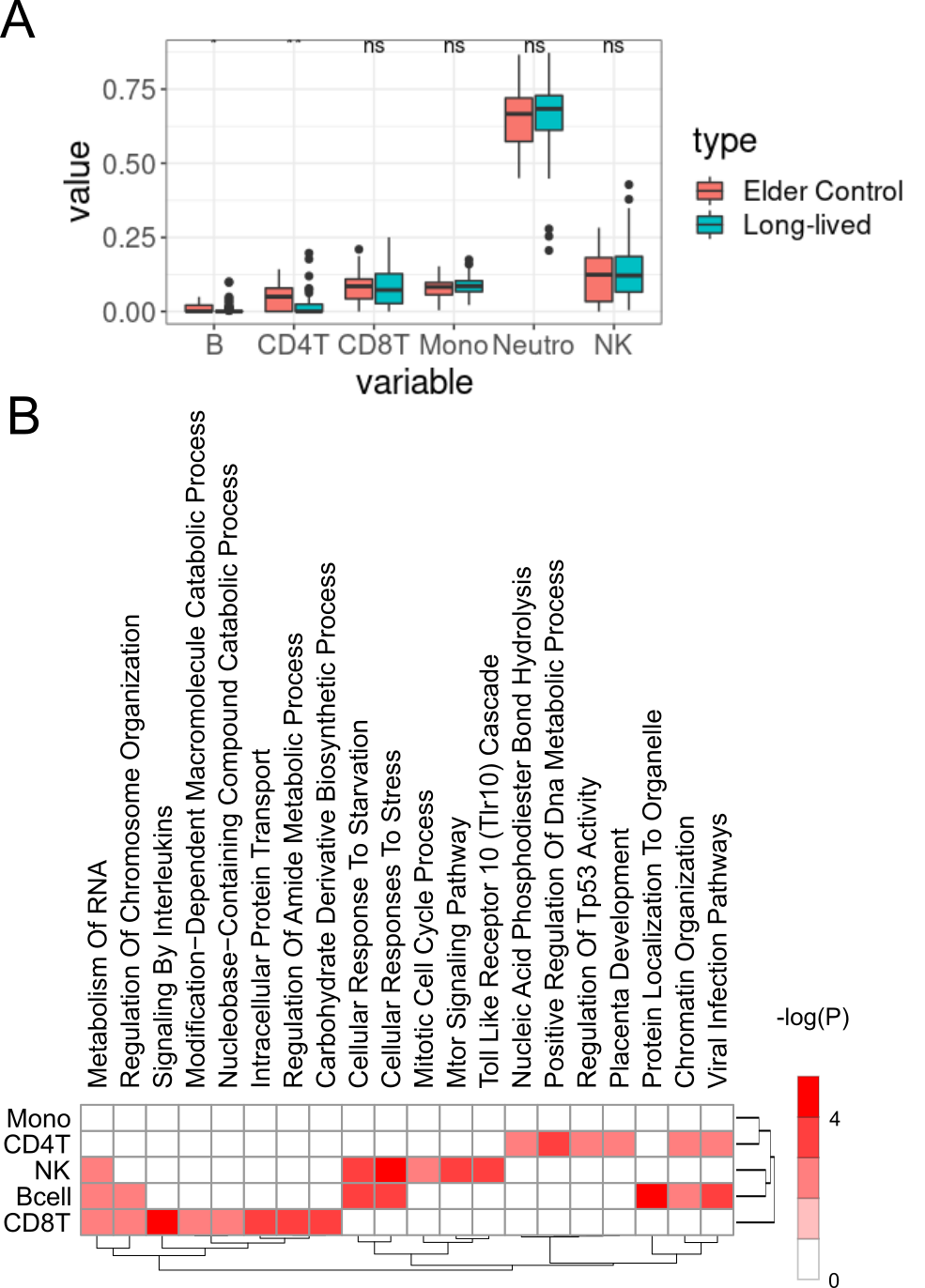


**Figure S6. Cell type-specific LLI-specific LERs. (A)** Methylome deconvoluted blood cell proportions estimated by EpiDISH**,** which was compared between groups by two-sided Wilcoxon’s rank sum test and significance values were plotted by asterisks (*p-value <0.05, **p-value <0.01). **(B)** Heatmap of enrichment results for genes whose promoters containing remaining five cell type-specific LLI-specific LERs.


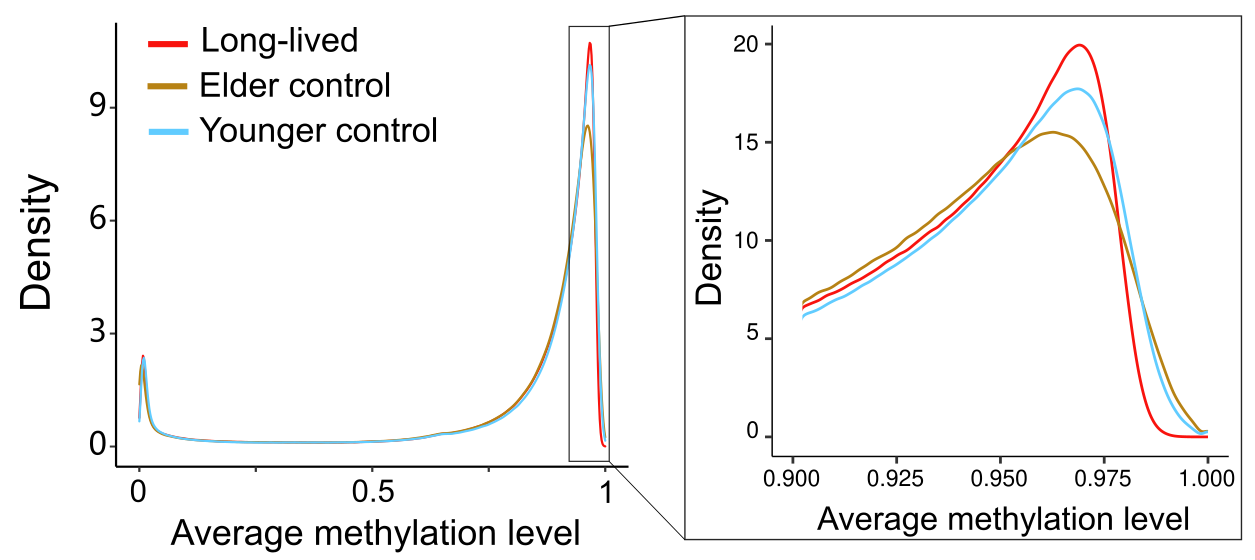


**Figure S7. Overall distribution of methylation levels in three age groups.**
